# On the margins of suicide: everyday horizons, turning points and trajectories of protection in peripheral young women

**DOI:** 10.1590/0102-311XEN055824

**Published:** 2024-11-04

**Authors:** Orli Carvalho da Silva, Joviana Quintes Avanci, Simone Gonçalves de Assis

**Affiliations:** 1 Instituto Nacional da Saúde da Mulher, da Criança e do Adolescente Fernandes Figueira, Fundação Oswaldo Cruz, Rio de Janeiro, Brasil.; 2 Escola Nacional de Saúde Pública Sergio Arouca, Fundação Oswaldo Cruz, Rio de Janeiro, Brasil.

**Keywords:** Self-injurious Behavior, Suicide, Minority Populations, Youth, Conducta Autodestructiva, Suicidio, Poblaciones Minoritarias, Juventud

## Abstract

Suicidal behavior and non-suicidal self-harm in vulnerable groups and population minorities pose a challenge for suicidology, complicating the universality of suicide. The goal of this paper is to analyze the lives of young women from marginalized communities, considering their experiences with suicidality and their relational and violent horizons. Nine women who took part in the fifth wave of a cohort on mental health and violence (2005-2022) in São Gonçalo, Rio de Janeiro State, Brazil, were interviewed (2022) about the contexts that kept them from committing suicide despite significant emotional distress from childhood through youth. From theme-based content analysis, three categories stood out and may contribute to an intersectional, decolonial and socially relevant approach to preventing self-destructive behavior. In the first, views on self-inflicted violence, better explained by the cores concepts of “sin” and “illness” than by the general violence they experienced. In the second, indirect references to self-harm behavior, where it was recognized that the use of euphemisms reflects not only the taboo but also the silencing of and discrimination against minorities. In the third, layers of protection and turning points, where “spirituality”, “occupation” and “motherhood” were interpreted as the main associations between factors of protection and resilience in the trajectories and daily lives of these young women. A closer look that acknowledges the humanity, rights and psychological distress of groups subjected to violence and discrimination not only enhances care and prevention of suicidal behavior but also deepens understanding of this human and universal phenomenon.

## Resumen

El comportamiento suicida y las autolesiones no suicida en grupos vulnerables y poblaciones minoritarias representan un desafío para la suicidología, lo que hace complexa la universalidad del suicidio. En este artículo, el objetivo fue analizar la vida de jóvenes de la periferia, considerando sus experiencias con el suicidio y sus horizontes relacionales y violentos. Nueve mujeres que participaron en la quinta ola de una cohorte sobre salud mental y violencia (2005-2022) en São Gonçalo, Estado de Río de Janeiro, Brasil, fueron entrevistadas (2022) sobre los contextos que las alejaron del suicidio, a pesar del importante sufrimiento emocional, desde la infancia hasta la juventud. Utilizando la técnica de análisis de contenido, modalidad temática, se destacaron tres categorías que pueden contribuir a un enfoque interseccional, decolonial y socialmente relevante para prevenir conductas autodestructivas. En el primero, opiniones sobre la violencia autoinfligida, mejor explicada por los núcleos “pecado” y “enfermedad”, y menos por la violencia vivida en general. En el segundo, lo casi dicho sobre las conductas autolesivas, en que se reconoció cómo el uso de palabras marginales sobre el tema refleja no solo el tabú, sino el silenciamiento y la discriminación contra las minorías. En el tercero, los niveles de protección y puntos de inflexión, donde la “espiritualidad”, el “trabajo” y la “maternidad” se interpretaron como las principales asociaciones entre los factores de protección y resiliencia en las trayectorias de vida y la vida cotidiana de estas jóvenes. Estrechar perspectivas, reconocer la humanidad, los derechos y el sufrimiento psicológico de grupos que han sido violados y discriminados, no solo califica el cuidado y la prevención de la conducta suicida, sino que amplía la comprensión de este fenómeno humano y universal.

## Introduction

Suicide has become a public health concern for global youth, a group in which this fatal outcome seems to provoke greater discomfort in micro and macro social domains, and where important elements of vulnerability are recognized ^1^ ^,^ ^2^ ^,^ ^3^. Recent global data suggest a non-uniform reduction in mortality rates, a reality that is not observed in the Americas or among young people from different minorities ^2^ ^,^ ^4^ ^,^ ^5^. In Brazil, there has been a significant increase in suicide and self-harm incidence rates among adolescents and young people in the past decade ^6^ ^,^ ^7^.

These differences guide a more detailed analysis of self-inflicted violence occurrence rates, in an effort to better understand these phenomena epidemiologically, clinically and socially, enabling interventions that can reduce early morbidity and mortality in this population ^2^ ^,^ ^8^. Such an analysis should involve elements that attempt to explain the particularities of youth suicidality among discriminated against minorities ^4^ ^,^ ^5^ ^,^ ^9^.

Giving importance to such characteristics means critically addressing suicidology, the field of study and debate on suicide, going beyond the biomedical perspective ^8^ ^,^ ^10^ ^,^ ^11^ ^,^ ^12^. Thus, the investigative focus on self-inflicted violence can transcend the individual sphere to reach the relational and systemic domains ^13^ ^,^ ^14^ ^,^ ^15^. Not just an individual in distress but a biological subject embedded in multiple relational and violent systems, whose development and subjectivity have been shaped by proximal (face-to-face) processes within specific socio-historical structures ^4^ ^,^ ^16^. This is recognized by the literature on self-inflicted violence and minorities ^13^ ^,^ ^17^ ^,^ ^18^ ^,^ ^19^ ^,^ ^20^, encouraging the consideration of counter-hegemonic and intersectional perspectives on psychological distress ^11^ ^,^ ^21^ and drawing attention to the importance of the relational and structural domains in the course of development, as proposed by Bronfenbrenner ^15^ and Bronfenbrenner & Ceci ^16^.

According to Silva Filho ^22^ suicidal behavior involves an overlap between psychological distress and violence (in its self-inflicted typology and in its physical and psychological forms). Emotional distress tends to be more evident and is viewed as a causal element that guides clinical practice ^8^ ^,^ ^23^. However, recognizing violence as an inherent element in this behavior expands the possibilities for understanding its occurrence, enabling reflections on and approaches to social structure and its impact on the course of life ^18^ ^,^ ^20^ ^,^ ^24^ ^,^ ^25^. The clash between these perspectives does not establish an etiological impasse but an overlap, where arguments are combined and the multiple causes of these phenomena are acknowledged ^8^ ^,^ ^22^.

Thus, different publications argue for the importance of recognizing violence as one of the factors determining self-harm behavior, especially when children, adolescents and youth are studied ^4^ ^,^ ^9^ ^,^ ^20^. In these cases, adverse childhood experiences gain prominence in qualitative-quantitative studies, corroborating concerns about the care of children and youth ^19^ ^,^ ^24^ ^,^ ^25^. This is not only for physical safety and monitoring growth but also for tracking developmental and mental health trajectories ^4^ ^,^ ^26^ ^,^ ^27^.

Suicidal behavior is viewed as a continuum that includes ideation, planning, attempts and completed suicide, a definition aligned with public health ^2^ ^,^ ^22^. Although there is no consensus in the literature, non-suicidal self-harm is not considered part of this spectrum, since its expression may resemble a suicide attempt but there is no accompanying ideation. Despite this delicate distinction, it can also be understood as a phenomenon involving self-inflicted violence and emotional distress ^8^ ^,^ ^28^. Both phenomena affect contemporary youth, requiring urgent reflection on development, mental health and violence ^1^ ^,^ ^3^ ^,^ ^26^ ^,^ ^28^.

In the face of so many feelings, behaviors and vulnerabilities, understanding why an individual does not give in to suicide becomes a potential path for prevention, especially among minorities with marginalized trajectories. The study of death guides reflection on life ^29^; thus, reflecting on young women on the borders of suicide may contribute to understanding their horizons, protective pathways and turning points. The goal of this paper is to discuss how young women from the outskirts of a violent Brazilian metropolis understand the suicidal behavior they exhibited throughout the course of their life, from childhood to youth, focusing on the events and contexts that protected them from self-inflicted death.

## Methodological approach

This paper is the result of a longitudinal study carried out with children in São Gonçalo, Rio de Janeiro State, Brazil, which began in 2005 ^30^. The reflections herein presented were developed from the qualitative analysis of nine interviews conducted in a complementary phase of the fifth investigative wave (2021-2022) of the *São Gonçalo/RJ Children’s Violence and Mental Health Cohort* (2005-2022).

São Gonçalo is located in the Metropolitan Area of Rio de Janeiro. It is a large and densely populated municipality with adverse living conditions, limited educational resources and poor infrastructure ^31^. It has numerous records of police operations, interventions with violent repercussions that reduce urban mobility, access and functionality ^32^. The presence of multiple vulnerabilities, in both micro and macro social contexts, is a characteristic of the cohort and a significant descriptor in monitoring these participants.

In 2021, 129 youngsters (25.8% of the initial sample) were located through in-person visits by community health workers (CHW), phone contact or on social media, and agreed to participate by answering questionnaires after signing an informed consent form. Of these 129 participants, 35 had at least one reference, over nearly two decades of the study, to suicidal behavior and/or non-suicidal self-harm. Given the theoretical and clinical divergence between suicidal behavior and non-suicidal self-harm ^8^ ^,^ ^23^, a conceptual alignment was adopted in this study, using the term suicidal behavior/non-suicidal self-harm. The rationale for this grouping was that both represent psychological distress and actions against bodily integrity.

Aiming at a deeper understanding, the qualitative method ^33^ was applied to a subset of these 35 participants. The onset of the outcome (before or after the age of 14) and the presence or absence of recurrence (more than one reference throughout the study) were additional criteria for recruitment in this phase. The age of 14 was chosen for marking the transition between the first and second halves of adolescence, with the latter being a typical phase of increased occurrence of suicidal behavior/non-suicidal self-harm ^2^ ^,^ ^4^ ^,^ ^6^ ^,^ ^28^. Recurrence is one of the most severe clinical elements concerning self-inflicted violence ^23^.

There were challenges in recruiting, scheduling and conducting the meetings with the selected young women, resulting in a final group of nine participants. The nine interviews were done either in person (at an easily accessible clinic) or remotely via the Zoom Meeting platform (https://zoom.us/) from September to October 2022. They were led by the paper’s author (O. C. Silva Filho; a psychiatrist) with the administration of a previously tested semi-structured questionnaire. The interview technique made it possible for relevant themes to come up and be discussed, respecting the individuality of each participant, with analysis of the accounts leading to thematic saturation ^34^. A second researcher (a psychologist) attended all the interviews, contributing to a supportive environment, which helped prevent any post-interview discomfort.

The young women received pseudonyms that refer to women who stand out in the defense of popular culture and human rights; thus, by honoring these women, we sought to make it difficult to identify the participants. The interviews were transcribed, creating a corpus that was examined according to theme-based content analysis ^33^ ^,^ ^34^ ^,^ ^35^. The transcribed material underwent repeated and progressively deeper readings in order to achieve comprehensive understanding, as well as to identify discourse similarities and differences ^34^. The most relevant excerpts from each interview were selected, tabulated and analyzed based on the theoretical framework of the investigation. The interview process revealed contradictions, repetitions and responses to the researchers’ questions, prompting reflection. This was followed by a synthesis of each interview, which made it possible to group them by theme and content. After being cross-checked and reorganized, they resulted in three stable analytical categories with interpretative significance, no longer tied strictly to the participants’ reports ^34^ ^,^ ^35^: (1) about views on self-inflicted violence; (2) about indirect references to self-harm behavior; (3) over protective layers and turning points. These categories were compared with the national and international literature, making up the results and discussion of this work ^33^.

All phases of the longitudinal study were approved by the Research Ethics Committee of the Sergio Arouca National School of Public Health, Oswaldo Cruz Foundation (ENSP/FIOCRUZ; CAAE 5734422.6.0000.5240).

## Results and discussion

The sociodemographic profile of the interviewed young women is shown in Box 1, characterized by nine marginalized women in a minority context who exhibited different characteristics of suicidal behavior/non-suicidal self-harm (Box 2).

**Box 1:** Sociodemographic profile of the interviewed young women (2022).

IDENTIFICATION	AGE (YEARS)/SKIN COLOR	MARITAL STATUS/ SEXUAL ORIENTATION/ CHILDREN (YEARS OF AGE)	EDUCATION/RESIDENCE	RELIGION	OCCUPATION
Teresa Cristina	25/White	Single/Heterosexual/No children	Complete high school/Lives with parents	*Candomblé*	Dance teacher
Leci Brandão	25/Black	Single/Heterosexual/1 daughter (7)	Incomplete high school/Lives with daughter	Neopentecostal evangelical	Seamstress, braider, manicurist
Conceição Evaristo	23/Black	Single/Heterosexual/ 2 children (7; 2)	Incomplete elementary education/Lives with mother and/or boyfriend	Neopentecostal evangelical	“Housewife”
Cora Coralina	24/Black	Single/Heterosexual/2 children (7; 4)	Incomplete elementary education/Lives with children, supported by parents	Neopentecostal evangelical (non-practicing)	Shopkeeper - on social support for medical reasons
Beth Carvalho	25/Black	Married/Heterosexual/No children	Complete higher education/Lives with husband	Neopentecostal evangelical	Business administrator
Elza Soares	24/Black	Single/Heterosexual/3 children (7; 4; 1)	Complete high school/Lives with daughters and partner	Neopentecostal evangelical	Braider
Clara Nunes	24/Black	Single/Heterosexual/1 daughter (8)	Complete high school/Lives with mother	No defined religion	Security
Jovelina Pérola	25/Black	Single/Heterosexual/2 children (9; 4), pregnant	Complete high school, incomplete nursing technician course/Lives with 2 children	No defined religion (former neopentecostal evangelical)	Hospital kitchen assistant - on social support for medical reasons (INSS)
Clementina de Jesus	24/Black	Single/Heterosexual/No children	Complete high school/Lives with boyfriend	No defined religion	Secretary

INSS: Brazilian Institute of Social Security.

Source: prepared by the authors.

**Box 2:** Characteristics of violence and types of interventions experienced throughout life, and categories of turning points identified in the interviews.

IDENTIFICATION	REPORTED VIOLENCE	CHARACTERISTICS	INTERVENTION AND LAYERS OF PROTECTION	TURNING POINTS
Teresa Cristina	School bullying, psychological violence and delinquency (drug trafficking) in the adolescence	Suicidal behavior (ideation and attempts) reported at age 11 and 14	Child Protection Council (rescue from delinquency and return to family), theater/dance classes (at school), religion (youth)	Arts, spirituality and legal device
Leci Brandão	Physical and psychological (childhood, adolescence and youth), sexual (childhood); deprivation throughout life	Suicidal behavior (ideation and attempts - childhood and adolescence) and non-suicidal self-harm (late adolescence) - reported at age 8, 11, 15, 24	No direct intervention, denies having asked for help. Throughout life, religion, motherhood and relationship with some adults of reference.	Spirituality and motherhood
Conceição Evaristo	Physical and psychological violence and deprivation (childhood, adolescence and youth); violence from intimate partner (youth); street fights	Suicidal behavior (ideation) and non-suicidal self-harm reported at age 15 and 24	No direct intervention. Children brought about a change in her life	Motherhood
Cora Coralina	Violence from intimate partner (youth), including pressing charges	Suicidal behavior (ideation) and non-suicidal self-harm reported at age 15 and 24	Psychotherapy, health care for her illness, reconnection with faith, legal intervention (Maria da Penha Law)	Spirituality, motherhood, psychotherapy/treatment and legal device
Beth Carvalho	Physical and verbal violence (childhood); psychological violence (childhood, adolescence and youth)	Suicidal behavior (ideation) and non-suicidal self-harm reported at age 8 and 15	Psychotherapy, extracurricular training, appreciation of faith	Spirituality and occupation, education
Elza Soares	Physical and psychological violence (childhood and adolescence); violence from intimate partner (adolescence)	Suicidal behavior and non-suicidal self-harm reported at age 23	Psychotherapy, use of psychotropic drugs, maintaining faith, braiding course/classes, pleasure in braiding	Occupation, motherhood, spirituality and psychotherapy/treatment
Clara Nunes	Psychological (childhood), physical (childhood and adolescence) violence	Suicidal behavior (ideation) and non-suicidal self-harm reported at age 10	Motherhood, work, sports (martial arts), motivational drive	Motherhood, occupation, sports and work
Jovelina Pérola	Physical and psychological violence (childhood, adolescence and youth); violence from intimate partner (adolescence and youth)	Suicidal behavior (ideation) and non-suicidal self-harm reported at age 9 and 24	Motherhood, study, psychotherapy	Motherhood, occupation, psychotherapy/treatment and education
Clementina de Jesus	Psychological violence (childhood and adolescence)	Suicidal behavior (ideation and attempts) and non-suicidal self-harm reported at age 14 and 23	Sport (self-defense, martial arts), study, spirituality	Occupation, spirituality, sports, education and work

Source: prepared by the authors.

### Nine peripheral women: overview of a minority

The interviewed young women showed different characteristics of suicidal behavior/non-suicidal self-harm (Box 2), being grouped as a female and peripheral group. No single minority is represented here; however, the group is exclusively composed of women born and living in peripheral areas of São Gonçalo. Such exclusivity ensured the inclusive nature of the study, with the nine women forming a minority marked by intersectionality or, as defined by Akotirene ^36^, by an interconnected system of oppressions.

Baére & Zanello ^10^ argue that gender roles in a patriarchal and violent society impact the mental health of women, to the point of questioning their emotional distress. In Brazil, women are predominant in cases of suicide attempts and non-suicidal self-harm, with the risk of suicide being higher among those aged 15 to 19 ^6^ ^,^ ^7^. These data highlight the relevance of sociocultural aspects in understanding suicidality ^10^ and concur with Jaworski’s ^37^ argument that for women, the focus of investigation should be the early stages of suicidal behavior rather than its fatal outcome.

The description of the young women as peripheral and marginalized goes beyond income or social class. The areas where the children were sampled were characterized by intense social vulnerability: marginalized, peripheral territories with high crime rates ^30^. It is important to note that despite possible convergences or associations between the concepts of marginality and criminality, this study follows the distinction made by Coelho ^38^ in describing the context of urban violence in Rio de Janeiro. For him, unemployment, underemployment or poverty serve as proxies for a marginalized population, elements that, while possibly facilitating the emergence of criminality, do not define it per se. At the same time, the recurrent and violent police operations in peripheral areas ^32^ corroborate the process of criminalizing marginality and marginalizing criminality ^38^.

Therefore, continuing to live and interact in these areas was understood as remaining immersed in the daily reality of violence and, consequently, vulnerability while growing up ^4^ ^,^ ^19^. Even while working and/or attending school, these young women were marginalized, victims of social inequality, with their lives and microsocial relationships shaped by structural violence ^15^.

Three categories were analyzed: (1) marginalized trajectories: views on self-inflicted violence; (2) euphemisms: indirect references to self-harm behavior; and (3) on the margin of suicide: layers of protection and turning points.

#### Marginalized trajectories: views on self-inflicted violence.

Theoretical and clinical arguments suggest that the overlap of different kinds of violence becomes a risk factor for its self-inflicted typology ^23^. This is certainly not exclusive to self-harm but it is a frequent condition in the study of violence ^28^. Among the interviewees, reports of violence can be grouped as follows: eight experienced family violence during childhood and adolescence; four faced violence at school; two reported sexual violence; and five experienced violence from an intimate partner.

According to the young women’s reports, their personal experiences with violence did not justify the presence of suicidal behavior/non-suicidal self-harm in their lives. This was initially surprising, as the consequences of violence are well-known in the physical, psychological, developmental and relational domains, especially for children and adolescents ^19^ ^,^ ^25^. The analysis did not dismiss this impact but pointed to a lack of recognition of the violence itself. The explanation went beyond the personal experiences of these young women, expanding to perceptions that reflected broader societal understandings of these phenomena.

Two explanatory core concepts prevailed throughout the analytical process: “sin” and “disease”. Both reflect the deviant nature of suicide in Western society, viewed as individual failings in or of human life. There were gradations within this category: “sin” and “disease” more easily related to completed suicide. As the most tragic outcome of the suicidal continuum, suicide was described as a “great sin” or “intense disease”, while non-suicidal self-harm was considered a deliberate act against one’s own integrity and a less severe flaw compared to suicide.

“*The enemy’s role is to kill, steal, destroy. So, he wants others to kill themselves. If they do so, they will have no salvation!*” (Leci Brandão).

There was a predominance of the neopentecostal perspective in the faith and value system of the interviewees. Four are practicing neopentecostals, one is non-practicing, and one is currently “removed”. The discourse of all of them was strongly marked by this faith. Even among the two young women “with no defined religion”, there was noticeable influence of neopentecostalism. This corroborates the findings of Cunha ^40^ who highlights the spread of this segment in the outskirts of Rio de Janeiro, especially after the 1990s, strongly reflected in the culture, daily life and language of this youth. Maia ^41^ also portrays this context, describing the visibility and everyday presence of young neopentecostals in São Gonçalo.

Thus, it was shown how neopentecostal beliefs and standards pervaded the trajectories of these young women, influencing their choices, shaping their perspectives and explaining their distress and self-harm practices. In this worldview, the world is in constant tension between good and evil ^41^, with suicidal ideation viewed by the interviewees as a malignant influence that makes them stray from the Lord’s path and promises.

“*There are times when we falter, when we sin. ‘Lord, I feel weak!’. Then, some bad thoughts come into our minds. We are not always fully connected to God*” (Cora Coralina).

From the perspective of “suicide as sin”, exercising faith is the main intervention to be sought when faced with the suicidal continuum, preventing suicidal ideation from escalating to an attempt. Ideation can be seen as a trial or temptation that, when overcome through faith, can honor one’s relationship with the sacred. Conversely, one of the failures of spirituality occurs in the face of suicide.

A religious framework that values simple sermons, with simple examples for simple people ^40^, has proved to be popular in peripheral areas, furthering the incorporation and spread of its principles, as identified in this corpus. Supported by prosperity and dominion theology ^40^ and the possibility of change in this life and not just in the afterlife ^41^, neopentecostalism gained muscle, playing a considerable role in shaping the horizons and trajectories of Brazilian youth.

Although this study acknowledges the plurality of neopentecostalism, such differences are not essential here, since they express a similar worldview that helps understand the micro and macro social relationships within this chronosystem ^15^. The aim is to incorporate them as a possible element in protection networks, accessing these spaces and institutions of neopentecostal culture and harnessing their potential as hubs for mental health care and prevention. This poses numerous challenges and conflicts, given the context of violence and conservatism, but it must be planned.

The explanation of suicide as a manifestation of illness was unanimous among the young women, a view strongly supported by the widespread diffusion of the biomedical model. According to this paradigm, about 80% to 90% of completed suicides have an associated mental disorder, a statistic often repeated in the literature ^6^ ^,^ ^23^. Thus, a convergence between common sense and hegemonic scientific knowledge was evident.

“*It’s a pain you carry, although you might not know it, but it’s a burden. You sleep with it, you wake up with it, you don’t want to think about it, but you end up thinking about it. It tortures you in such a way. I often say that I don’t judge those who want to take their own life*” (Elza Soares).

This outlook does not seem to represent a weakening of taboos surrounding child and adolescent suicide ^41^ but rather the result of campaigns addressing suicide prevention through health. A Brazilian suicide prevention campaign was mentioned by several of the young women, suggesting that it had become part of their everyday lives, especially through social media. However, such embedment does not necessarily lead to significant individual or collective changes in prevention, often being a presence they described as indifferent or tedious.

The reports of the participants emphasized, as supported by biomedical literature, a possible connection between depression and suicidal behavior ^1^ ^,^ ^2^ ^,^ ^23^, with examples of how psychological distress can lead to the occurrence of this violence. Two elements were identified as supporting this view: four of the nine young women had undergone psychotherapy; and at certain times the reports overlapped with descriptions of intense sadness and hopelessness. Although the investigation was not a diagnostic interview, the theoretical framework of suicidology made it possible to reach these conclusions in the analysis.

Eight young women reported symptoms consistent with mental disorders throughout their lives, but only four had access to psychotherapy and none underwent psychiatric treatment, with cases of irregular use of psychotropic drugs. Thus, personal experiences of emotional distress, even if not reduced to a mental disorder, could serve as an argument for the correlation between suicidal behavior and mental illness.

“*It was more of a ‘I’m tired!’ situation. It was a feeling of: ‘Ah, I’ve had enough, I don’t want to live anymore. I’m tired. It only brings problems, it only brings stress, nothing gets better, I don’t see anything happening, it only brings sadness’. I would think that it would be easier to end it*” (Clementina de Jesus).

It should be noted that these young women had little support to deal with their psychological distress. It was possibly recognized but not addressed due to poor access to psychosocial support networks, or, even worse, was not validated as distress at all. Alvarez et al. ^4^ point out that only one-third of African American youth who die by suicide have a diagnosed mental disorder. Baére & Zanello ^10^ and Jaworski ^37^ illustrate how the lower lethality of suicide attempts among women may be socially perceived as indicating less suffering compared to men. Navasconi ^11^, in a critical stance to the biomedical perspective, argues that racism is a factor in illness and suicide among Brazilian youth. These arguments come from different authors with varying theoretical perspectives, which, despite their affiliations, converge on the need to consider prejudice against minorities in the management and prevention of suicidal behavior and non-suicidal self-harm.

#### Euphemisms: indirect references to self-harm behavior

All interviews were preceded by a brief explanation of the research, identifying its subject and motivation. This helped in understanding the difficulty observed to speak in the first person and in using words and terms directly related to suicidal behavior/non-suicidal self-harm.

Speaking about third parties, whether known or not, was constant, allowing the young women to expand their arguments, examples and causal explanations for self-inflicted violence. Similarly, it seemed easier to resort to metaphors or illustrations to narrate and exemplify their own experiences. At this point, a comprehensive leap beyond what was actually expressed made it possible to listen to and interpret what was implied or silenced ^33^.

“*My mother would beat me and I wanted to disappear from her life. I didn’t want to die, but I wanted to die for her because she wouldn’t see me anymore. She wouldn’t even know I existed anymore. I wanted to disappear, almost die*” (Clara Nunes).

“*I don’t know, I think I’ve never wanted to kill myself. I wanted to disappear, to hide, to crawl into a hole and stay there hidden, without anyone seeing me. To disappear and if there was a solution, I would come back*” (Conceição Evaristo).

“*It was as if I were lying on a stretcher in the hospital. I was lying there, I could the doctor, but I couldn’t see anyone from my family. I would feel despondent. ‘Could this be really true? No-one? It’s not possible’. Then I saw that no one showed up. I’d get a little nervous, stressed, thoughtful*” (Elza Soares).

More than identifying and even understanding what was implied by the young women, incorporating these euphemisms into the semantics of care is one of the possibilities of this research. It was considered that the crystallization of the taboo surrounding child and youth suicide ^42^ might have been a reason for avoiding the subject. This taboo blocks the subject, silencing and constraining discourse. However, the personal narratives presented alongside the contemporary prominence given to the subject suggest that other elements might clarify these indirect references.

Drawing on studies that highlight discrimination against minorities as a risk factor for suicidal behavior/non-suicidal self-harm ^19^ ^,^ ^20^, it is considered that the lack of social validation of psychological distress in the course of these young women’s lives is a sensitive and pertinent understanding of this empirically observed issue. Non-validation or inadequate handling of a distress, which is only acknowledged, addressed and managed according to hegemonic practices and norms, as discussed by Fanon ^21^.

Benton ^5^ points to a greater number of suicide attempts than reports of suicidal ideation among black youth, corroborating the idea that that the suffering of this minority is only validated when extreme action is taken. It is as if psychological distress were naturalized and prohibited in minority groups, not acknowledged. At the same time, Jaworski ^37^ stresses that the lower global suicide rates among women should not obscure the presence of suicidal ideation within this gender, drawing attention to other forms of discrimination.

Sheftall et al. ^9^ argue that structural and relational violence should guide investigations, approaches and preventive actions for suicidality among minority youth. In Brazil, Navasconi ^11^ denounces how the scientific literature and health care ignore the intersectionality of suicidal behavior with markers of race, class, and gender.

Given that different forms of discrimination and violence are present in the social relations of peripheral groups, especially those involving women, it is essential that discussions about suicidality consider environmental adversities and adverse childhood experiences ^19^, addressed through sensitive listening and viewed through the lenses of justice, equity, diversity and inclusion. The repeated and naturalized violence against these groups amounts to daily micro aggressions that impact their mental health, especially when they are silenced or ignored ^27^.

In this context, the more detached discourse of the interviewed young women about suicidal behavior/non-suicidal self-harm did not seem to reflect mere discomfort at being interviewed. The implied words appeared to reveal a suppression of distress, or even to indicate lost opportunities for support in the face of different kinds of violence (Box 2) within relational systems throughout their life trajectories.

#### On the margin of suicide: layers of protection and turning points

This category made it possible to recognize the elements of the young women’s everyday life (social functions and/or interventions) identified as layers of protection against suicide (Box 2). These elements were grouped into eight first-order categories, which were then interpreted and reclassified into three second-order categories ^33^. The three categories - spirituality, occupation and motherhood - were perceived as the most significant turning points in the experiences of the young women. This synthesis provided insight into the contexts that enabled a trajectory that kept them on the margin of suicide, despite their life experiences of suicidal behavior/non-suicidal self-harm.

Highlighting these turning points was also a methodological resource used by Werner & Smith ^43^ in a cohort of Hawaiian children (United States) that began in the 1950s. The authors sought to understand, in adulthood, which everyday elements had been longitudinally protective. Higher education; skills learned during military service; stable marriage; participation in a faith community; recovery from a life-threatening event; and psychotherapy were recognized as protective elements in the Hawaiian cohort. Some of these elements were similar to those found in this study.

The prior discussion about the influence of spirituality in the lives of the young women justifies the importance of the “spirituality” category as an element of protection and resilience. More than an ontological consideration, its relevance herein lies in its ordinary concreteness: spirituality provides organization and inclusion in a support network in real everyday life. Organic groups that come together, validating values and striving for a better life ^40^. The individual choice of religious engagement during adolescence showed greater appeal as social support than mere continuation of a religious family tradition ^41^.

In this study, “occupation” does not relate merely to work. Given that labor capacity is a parameter for evaluating mental disorders and suicidality as deviations from the social order ^39^, this dimension was carefully considered beyond the term “work”. The occupation category included other interventions and protective layers (Box 2): arts (dance teaching course), pleasure in braiding (braiding course), level of education and actual work practice (integration into the social fabric). The young women confirmed that their ability - whether acquired or desired - to provide for themselves affords them security and dignity. As peripheral women, the pursuit of financial independence is a clear protective factor, given the violence and emotional distress they face, especially for those with children.

Education, a formative element of this category, was a focal point of criticism, even though only two young women had not completed elementary school. School did not prove to be a space of adequate development for their daily lives and failed to provide support in the face of violence, establishing itself as a violent environment. Regarding suicidal behavior/non-suicidal self-harm, the accounts predominantly indicated an inability to engage in dialogue and management. In this context, Sheftall et al. ^9^ argue that black youth tend to feel less secure regarding mental health in school and do not seek as much help as their peers. School support systems do not reach everyone, requiring strategies that are more sensitive to the subjectivity of minority groups. The authors ^9^ believe that training community leaders and religious organizations in violence intervention, conflict management and recognition of emotional distress could help spread, in a contextualized and decolonial manner, strategies for preventing and addressing suicidal behavior/non-suicidal self-harm, giving voice and agency to historically silenced and disempowered identities, as argued by Ribeiro ^44^.

Motherhood was an important everyday factor: of the nine young women, six were mothers, with one in her third pregnancy; of the three without children, one expressed the wish to be a mother. At first glance, the presence of motherhood might seem to represent the perpetuation of the female role in procreation, whether desired or not, or even be a choice for these young women. However, the reports said otherwise. For some of the participants, pregnancy negatively impacted their life course, leading to school dropout, increased violence and worsening of psychological distress.

When asked how motherhood influenced suicidal behavior/non-suicidal self-harm, all agreed that pregnancy and childbirth were protective elements, even for those who reported distress and violence. According to the young women, motherhood was mainly an opportunity to recount their own stories, to rethink values and attitudes: an experience of change. The romanticized tone in this perspective was questioned, showing that their children became their greatest concern when they became mothers. Their children, especially the daughters, needed to be protected from the violence they had experienced. This required significant effort, resulting in life planning that led them away from suicidality.

“*I have a daughter. I need to show her that this is not the way to go. I don’t need to ruin my life because if I do, my daughter will also ruin hers. Can you imagine that?*” (Leci Brandão).

Not that motherhood is universally protective, especially given the possibility of worsening violence and mental disorders ^23^. However, it is important to recognize that motherhood may lead to reorganization in social relationships and provide hope; a hopeful horizon is a point of protection.8 At the same time, transgenerational concerns regarding gender violence should be considered ^10^, in which fear for the future of daughters is greater than for sons.

“*This person here is a warrior, fighting for her daughters, but daily life is sad, it has become sad, something I can’t even talk about. I look in the mirror and say, ‘Why am I crying? Why am I sad? I have my daughters, I have my home!’*” (Elza Soares).

It is stressed that turning points can be conceived not only as factors or layers of protection but as an association between them and elements of resilience ^43^, which are important due to their specificity and historicity, according to the bioecological model ^15^. It is argued that understanding suicidality and planning psychosocial interventions should consider life trajectories, the community and the significant elements in the everyday life of individuals and their peers ^11^. A critical approach to community space is not presented here as an innovation, as it is an essential element of public health. However, suicidology views this practice as inadequate, especially concerning minorities, whose marginalized existences tend to be less addressed and understood by the different fields of knowledge and public policies, as highlighted by Lima & Navasconi ^13^ and Ribeiro ^44^.

## Conclusions

In this paper, life was examined through experiences of suicidality reported by peripheral youth. Life and death are intertwined in a dialogue marked by distress and violence that shape the subjectivity and horizon of marginalized youth. Addressing such distress from experiences at the border of death (ideation and suicide attempts) and non-suicidal self-harm enables a dynamic view of risk and, more importantly, of protection for these minority groups, whose suffering and expressions are often less validated, receiving less support and psychosocial interventions. This perspective also highlights the impact of gender violence on the relational horizon of women.

The reality of peripheral women as a minority group was explored to reflect on suicidal behavior/non-suicidal self-harm; however, the discussion extended beyond gender and social class. One limitation of this study is that its arguments overlap different minorities, although this may strongly contribute to counter-hegemonic and intersectional discussions on suicidality among youth in the Global South. Further investigations into the relationship between suicidal behavior and urban violence, or the nuances of ethnic-sexual minorities, could complement the reflections presented here. Another potential limitation was that information on suicidal behavior/non-suicidal self-harm in the early waves of the cohort was obtained from parents/guardians and teachers, although they are important sources in child mental health research.

Diversifying clinical and epidemiological settings should be on the agenda for new suicidology research, urgently and critically acknowledging that the phenomenon of suicide does not solely comprise major and hegemonic groups. The human and the universal are filled with minorities, with their specificities and complexities; by acknowledging this, it may become more feasible to reduce morbidity and mortality from suicidal behavior among youth. Looking more closely and deeply at abused groups not only enhances care and prevention of suicidal behavior in this population but also expands understanding of the phenomenon. However, this requires recognizing the humanity, rights and mental health of historically vulnerable and marginalized groups.

At the same time, reflecting on the resilience of life and of these female bodies means acknowledging the existence of protective strategies that go beyond biomedical care. Layers of protection and turning points are established in the daily life of youth, where and with whom they move about, interact, practice their beliefs, and live out their desires and work. Transforming these contexts into “factors of protection” may mean intervening from the viewpoint of equity, intersectionality and inclusion, extrapolating from individual to structural strategies. Thus, preventing self-inflicted violence should validate, in an ethical and decolonial practice, the subjectivities, knowledge and distress that are erased and neglected.
