# Supplementary material for: On the margins of suicide: everyday horizons, turning points and trajectories of protection in peripheral young women
Source: Cad Saude Publica. 2024 Nov 4;40(10):e00055824. doi: 10.1590/0102-311XEN055824 (PMC11540280; doi:10.1590/0102-311XEN055824)
Supplement: Translation

# Translation: À margem do suicídio: horizontes cotidianos, pontos de virada e trajetórias de proteção em jovens mulheres periféricas

**DOI:** 10.1590/0102-311XPT055824

**Authors:** Orli Carvalho da Silva, Joviana Quintes Avanci, Simone Gonçalves de Assis

**Affiliations:** 1 Instituto Nacional da Saúde da Mulher, da Criança e do Adolescente Fernandes Figueira, Fundação Oswaldo Cruz, Rio de Janeiro, Brasil.; 2 Escola Nacional de Saúde Pública Sergio Arouca, Fundação Oswaldo Cruz, Rio de Janeiro, Brasil.

**Keywords:** Comportamento Autodestrutivo, Suicídio, Minorias Populacionais, Juventude

## Abstract

Comportamento suicida e autolesões não suicida em grupos vulneráveis e minorias populacionais representam um desafio para a suicidologia, complexificando a universalidade do suicídio. Neste artigo, objetivou-se analisar a vida de jovens periféricas considerando suas experiências com a suicidalidade e seus horizontes relacionais e violentos. Nove mulheres participantes da quinta onda de uma coorte sobre saúde mental e violência (2005-2022) em São Gonçalo, Rio de Janeiro, Brasil, foram entrevistadas (2022) sobre os contextos que as mantiveram à margem do suicídio, apesar de importante sofrimento emocional, da infância à juventude. A partir da técnica de análise de conteúdo, modalidade temática, três categorias se destacaram e podem colaborar para uma abordagem interseccional, decolonial e socialmente relevante para prevenção do comportamento autodestrutivo. Na primeira, as visões sobre a violência autoinflingida, mais bem explicadas pelos núcleos “pecado” e “doença”, e menos pelas violências experimentadas em geral. Na segunda, o quase dito sobre comportamento autolesivo, em que se reconheceu como o uso de palavras marginais sobre o tema reflete não apenas o tabu, mas o silenciamento e a discriminação contra minorias. Na terceira, as camadas de proteção e pontos de virada, onde “espiritualidade”, “ofício” e “maternidade” foram interpretados como as principais associações entre fatores de proteção e resiliência nas trajetórias de vida e cotidiano dessas jovens. Estreitar olhares, reconhecendo a humanidade, os direitos e o sofrimento psíquico de grupos violentados e discriminados, não apenas qualifica o cuidado e a prevenção do comportamento suicida, mas amplia o entendimento desse fenômeno humano e universal.

## Introdução

O suicídio se tornou uma preocupação da Saúde Pública para a juventude global, grupo no qual esse desfecho letal parece provocar maior desconforto nas esferas micro e macrossociais, e em que se reconhecem importantes elementos de vulnerabilidade ^1^ ^,^ ^2^ ^,^ ^3^. Dados mundiais recentes indicam a redução não uniforme nas taxas de mortalidade, realidade não encontrada no continente americano ou em jovens de diferentes minorias ^2^ ^,^ ^4^ ^,^ ^5^. No Brasil, houve crescimento significativo nas taxas de incidência de suicídio e de autolesões em adolescentes e jovens na última década ^6^ ^,^ ^7^.

Tais variações orientam a desagregação das taxas de ocorrência das violências autoprovocadas, num esforço para melhor compreender epidemiológica, clínica e socialmente esses fenômenos, permitindo intervenções capazes de reduzir a morbimortalidade precoce dessa população ^2^ ^,^ ^8^. Essa desagregação deve envolver elementos que expliquem as diferentes apresentações da suicidalidade na juventude em relação a minorias discriminadas ^4^ ^,^ ^5^ ^,^ ^9^.

Dar relevância a tais características é abordar criticamente a suicidologia, o campo de estudo e o debate sobre suicídios, indo além da perspectiva biomédica ^8^ ^,^ ^10^ ^,^ ^11^ ^,^ ^12^, de forma que o olhar investigativo sobre as violências autoinfligida extrapole os domínios individuais e chegue aos relacionais e sistêmicos ^13^ ^,^ ^14^ ^,^ ^15^. Não apenas um indivíduo em sofrimento, mas um sujeito biológico inserido em múltiplos sistemas relacionais e de violência, cujo desenvolvimento e a subjetividade se construíram mediados por processos proximais (face-a-face) em estruturas sócio-históricas específicas ^4^ ^,^ ^16^. Isso é reconhecido pela literatura sobre violência autoprovocada e minorias ^13^ ^,^ ^17^ ^,^ ^18^ ^,^ ^19^ ^,^ ^20^, promovendo a consideração de perspectivas contra-hegemônicas e interseccionais em relação ao sofrimento psíquico ^11^ ^,^ ^21^, num destaque para a importância do domínio relacional e estrutural no curso do desenvolvimento, como proposto por Bronfenbrenner ^15^ e Bronfenbrenner & Ceci ^16^.

Segundo Silva Filho ^22^, o comportamento suicida é uma sobreposição de sofrimento psíquico e de violência (em sua tipologia autoinfligida e nas naturezas física e psicológica). O sofrimento emocional tende a ser mais evidente, sendo alocado como elemento causal, orientando a clínica ^8^ ^,^ ^23^. Porém, o reconhecimento da violência como elemento inerente nesse comportamento amplia as possibilidades de entendimento da sua ocorrência, permitindo reflexões e abordagens sobre a estrutura social e seu impacto no curso da vida ^18^ ^,^ ^20^ ^,^ ^24^ ^,^ ^25^. O confronto entre tais perspectivas não estabelece um impasse etiológico, mas uma sobreposição, em que se somam argumentos e se reconhece a multicausalidade desses fenômenos ^8^ ^,^ ^22^.

Assim, diferentes publicações defendem a pertinência no reconhecimento das violências como um dos fatores que determina as práticas autolesivas, principalmente quando crianças, adolescentes e jovens são estudados ^4^ ^,^ ^9^ ^,^ ^20^. Nesses casos, as experiências adversas na infância ganham relevância em estudos qualiquantitativos, corroborando a preocupação sobre o cuidado infantojuvenil ^19^ ^,^ ^24^ ^,^ ^25^. Não apenas para a segurança física e na vigilância do crescimento, mas para o seguimento de trajetórias desenvolvimentais e em saúde mental ^4^ ^,^ ^26^ ^,^ ^27^.

Por comportamento suicida, considera-se o continuum que envolve a ideação, o planejamento, as tentativas e o suicídio consumado, definição filiada à saúde pública ^2^ ^,^ ^22^. De forma não consensual na literatura, as autolesões não suicida não compõem esse espectro, ainda que sua expressão possa se assemelhar a uma tentativa de suicídio não acompanhada de ideação. Apesar dessa delicada distinção, também podem ser compreendidas como um fenômeno que envolve violência autoprovocada e sofrimento emocional ^8^ ^,^ ^28^. Ambos impactam a juventude contemporânea, demandando reflexões urgentes sobre desenvolvimento, saúde mental e violência ^1^ ^,^ ^3^ ^,^ ^26^ ^,^ ^28^.

Diante de uma multiplicidade de expressões de sentimentos, comportamentos e vulnerabilidades, o motivo de não sucumbir ao suicídio torna-se um caminho potencial de prevenção; principalmente em minorias cujas trajetórias estão à margem da vida. O estudo da morte orienta reflexões sobre a vida ^29^; assim, refletir sobre as jovens nas fronteiras do suicídio pode colaborar na compreensão de seus horizontes, percursos de proteção e pontos de virada. Neste artigo, objetivou-se discutir como jovens da periferia de uma violenta metrópole brasileira compreendem o comportamento suicida que apresentaram em suas trajetórias de vida, da infância à juventude, em destaque para os eventos e contextos que as protegeram da morte autoprovocada.

## Metodologia

Este artigo é fruto de estudo longitudinal com crianças em São Gonçalo, Rio de Janeiro, Brasil, iniciado em 2005 ^30^. As reflexões apresentadas foram construídas a partir da análise qualitativa de nove entrevistas realizadas em uma fase complementar da quinta onda investigativa (2021-2022) da *Coorte Violência e Saúde Mental de Crianças de São Gonçalo/RJ* (2005-2022).

São Gonçalo se localiza na Região Metropolitana do Rio de Janeiro, sendo um extenso e populoso município onde predominam condições de vida adversas, poucos recursos educacionais e uma infraestrutura precária ^31^. Apresenta muitos registros de operações policiais, intervenções com repercussões violentas que reduzem a mobilidade, o acesso e o funcionamento urbanos ^32^. A presença de múltiplas vulnerabilidades, em contexto micro e macrossocial, é um caracterizador da coorte, sendo descritor relevante no seguimento desses participantes.

Cento e vinte e nove jovens (25,8% da amostra inicial) foram localizados em 2021 por meio de visitas presenciais de agentes comunitários de saúde (ACS), contato telefônico ou em redes sociais, e aceitaram participar, respondendo questionários, mediante assinatura de Termo de Consentimento Livre e Esclarecido (TCLE). Desses 129 participantes, 35 têm ao menos uma referência, ao longo das quase duas décadas de estudo, a comportamento suicida e/ou autolesão não suicida. Nesta pesquisa, diante de divergência teórica e clínica entre comportamento suicida e autolesão não suicida ^8^ ^,^ ^23^, deliberou-se por aproximação conceitual, utilizando-se a expressão comportamento suicida/autolesão não suicida. Entendeu-se que esse agrupamento se justifica por ambas representarem desconforto psíquico e ação contra a integridade corporal.

Em busca de um aprofundamento compreensivo, o método qualitativo ^33^ foi aplicado em parte desses 35 participantes. O início de aparecimento do desfecho (antes ou após 14 anos de idade) assim como a presença ou a ausência de sua recorrência (mais de uma referência ao longo da investigação) foram os critérios adicionais no recrutamento dessa etapa. A idade de 14 anos foi escolhida por representar um marco etário entre as metades da adolescência, sendo a última uma típica fase de aumento de ocorrência de comportamento suicida/autolesão não suicida ^2^ ^,^ ^4^ ^,^ ^6^ ^,^ ^28^. A recorrência é um dos elementos clínicos de maior gravidade diante das violências autoinfligidas ^23^.

Houve dificuldade para o convite, o agendamento e a realização dos encontros com as jovens selecionadas, chegando-se a nove participantes. As nove entrevistas foram realizadas presencialmente, num consultório em região de fácil acesso, ou remotamente, por meio da plataforma Zoom Meeting (https://zoom.us/), entre setembro e outubro de 2022. Foram conduzidas pelo autor do artigo (O. C. Silva Filho; psiquiatra), a partir de um roteiro semiestruturado e testado preliminarmente. A técnica de entrevista permitiu o surgimento e o aprofundamento dos temas de interesse, respeitando a peculiaridade de cada jovem, com relatos cuja análise levou à saturação temática ^34^. Um segundo pesquisador (psicólogo) acompanhou todas as entrevistas, contribuindo para um ambiente acolhedor, o que colaborou para a ausência de mal-estar posterior.

As jovens receberam pseudônimos que fazem referência a mulheres que se destacam na defesa da cultura popular e dos direitos humanos; buscou-se, assim, homenageando essas mulheres, dificultar a identificação das participantes. As entrevistas foram transcritas produzindo um corpus examinado pela técnica de análise de conteúdo chamada de modalidade temática ^33^ ^,^ ^34^ ^,^ ^35^. O material transcrito passou por leituras seriadas e progressivamente aprofundadas, buscando-se uma compreensão global, assim como semelhanças e diferenças discursivas ^34^. Foram selecionados e tabulados os trechos mais relevantes de cada entrevista, analisados a partir do arsenal teórico da investigação. O processo de entrevistas desvelou contradições, repetições e respostas a questionamentos numa provocação do campo aos pesquisadores. Seguiu-se para etapa de síntese de cada entrevista, permitindo agrupamentos temáticos e de conteúdo, confrontados e reagrupados, produzindo três categorias analíticas estáveis, de cunho interpretativo, não mais aprisionadas às falas ^34^ ^,^ ^35^: (1) sobre as visões sobre a violência autoinfligida; (2) a respeito do quase dito sobre o comportamento autolesivo; (3) sobre as camadas de proteção e pontos de virada. Essas categorias foram confrontadas com a literatura nacional e internacional, compondo os resultados e a discussão deste trabalho ^33^.

Todas as etapas do estudo longitudinal receberam aprovação pelo Comitê de Ética em Pesquisa da Escola Nacional de Saúde Pública Sergio Arouca, Fundação Oswaldo Cruz (ENSP/FIOCRUZ; CAAE 5734422.6.0000.5240).

## Resultados e discussão

O perfil sociodemográfico das jovens entrevistadas é apresentado no Quadro 1, caracterizado por nove mulheres periféricas num panorama de minoria que apresentaram diferentes características do comportamento suicida/autolesão não suicida (Quadro 2).

**Quadro 1:** Perfil sociodemográfico das jovens entrevistadas (2022).

IDENTIFICAÇÃO	IDADE (ANOS)/COR DA PELE	ESTADO CIVIL/ ORIENTAÇÃO SEXUAL/ FILHOS (IDADE EM ANOS)	ESCOLARIDADE/RESIDÊNCIA	RELIGIÃO	OCUPAÇÃO
Teresa Cristina	25/Branca	Solteira/Heterossexual/Sem filhos	Ensino Médio completo/Reside com os pais	Candomblé	Professora de dança
Leci Brandão	25/Preta	Solteira/Heterossexual/1 filha (7)	Ensino Médio incompleto/Reside com a filha	Evangélica neopentecostal	Costureira, trancista, manicure
Conceição Evaristo	23/Preta	Solteira/Heterossexual/2 filhos (7; 2)	Ensino Fundamental incompleto/Reside com a mãe e/ou namorado	Evangélica neopentecostal	“Do lar”
Cora Coralina	24/Preta	Solteira/Heterossexual/2 filhos (7; 4)	Ensino Fundamental incompleto/Reside com filhos, com suporte dos pais	Evangélica neopentecostal (não praticante)	Comerciante - em licença médica (INSS)
Beth Carvalho	25/Preta	Casada/Heterossexual/Sem filhos	Ensino Superior completo/Reside com marido	Evangélica neopentecostal	Administradora
Elza Soares	24/Preta	Solteira/Heterossexual/3 filhos (7; 4; 1)	Ensino Médio incompleto/Reside com filhas e companheiro	Evangélica neopentecostal	Trancista
Clara Nunes	24/Preta	Solteira/Heterossexual/1 filha (8)	Ensino Médio completo/Reside com a mãe	Sem religião definida	Vigilante
Jovelina Pérola	25/Preta	Solteira/Heterossexual/2 filhos (9; 4), gestante	Ensino Médio completo; técnico de enfermagem incompleto/Reside com 2 filhos	Sem religião (evangélica neopentecostal afastada)	Copeira em hospital - em licença médica (INSS)
Clementina de Jesus	24/Preta	Solteira/Heterossexual/Sem filhos	Ensino Médio completo/Reside com o namorado	Sem religião definida	Secretária

INSS: Instituto Nacional do Seguro Social .

Fonte: elaboração própria.

**Quadro 2:** Características das violências e modalidades de intervenções experimentadas ao longo da vida e categorias dos pontos de virada encontradas nas entrevistas.

IDENTIFICAÇÃO	VIOLÊNCIAS REFERIDAS AO LONGO VIDA	CARACTERÍSTICAS	INTERVENÇÕES E CAMADAS DE PROTEÇÃO	PONTOS DE VIRADA
Teresa Cristina	*Bullying* escolar, violência psicológica e delinquência (tráfico drogas) na adolescência	Comportamento suicida (ideação e tentativas) relatado aos 11 e 14 anos	Conselho Tutelar (resgate delinquência e retorno familiar), aulas de teatro/dança (pela escola), religião (juventude)	Artes, espiritualidade e dispositivo legal
Leci Brandão	Física e psicológica (infância, adolescência e juventude), sexual (infância); privações ao longo da vida	Comportamento suicida (ideação e tentativas - infância e adolescência) e autolesões não suicida (final da adolescência) - relatados aos 8, 11, 15, 24 anos	Não houve uma intervenção direta, nega ter solicitado ajuda. Ao longo da vida, religião, maternidade e convívio com alguns adultos de referência	Espiritualidade e maternidade
Conceição Evaristo	Violência física, psicológica e privações (infância, adolescência e juventude); violência entre parceiro íntimo (juventude); brigas na rua	Comportamento suicida (ideação) e autolesões não suicida relatado aos 15 e 24 anos	Não houve uma intervenção direta. Os filhos trouxeram mudança de vida	Maternidade
Cora Coralina	Violência entre parceiro íntimo (juventude), incluindo denúncia	Comportamento suicida (ideação) e autolesões não suicida relatados aos 15 e 24 anos	Psicoterapia, cuidados em saúde por sua doença, reaproximação da fé, intervenção legal (Lei Maria da Penha)	Espiritualidade, maternidade, psicoterapia/tratamento e dispositivo legal
Beth Carvalho	Violência física e verbal (infância); violência psicológica (infância, adolescência e juventude)	Comportamento suicida (ideação) e autolesões não suicida relatados aos 8 e 15 anos	Psicoterapia, formação extracurricular, valorização da fé	Espiritualidade, ofício e educação
Elza Soares	Violência física e psicológica (infância e adolescência); violência entre parceiro íntimo (adolescência)	Comportamento suicida e autolesões não suicida relatados aos 23 anos	Psicoterapia, uso de psicofármacos, manutenção da fé, curso/aulas de trancista, prazer em trançar	Ofício, maternidade, espiritualidade e psicoterapia/tratamento
Clara Nunes	Violências psicológica (infância), física (infância e adolescência)	Comportamento suicida (ideação) e autolesões não suicida relatados aos 10 anos	Maternidade, trabalho, esporte (artes marciais), ímpeto motivacional	Maternidade, ofício, esporte e trabalho
Jovelina Pérola	Violências física e psicológica (infância, adolescência e juventude); violência entre parceiro íntimo (adolescência e juventude)	Comportamento suicida (ideação) e autolesões não suicida relatado aos 9 e 24 anos	Maternidade, estudo, psicoterapia	Maternidade, ofício, psicoterapia/tratamento e educação
Clementina de Jesus	Violências psicológica (infância e adolescência)	Comportamento suicida (ideação e tentativas) e autolesões não suicida relatados aos 14 e 23 anos	Esporte (defesa pessoal, artes marciais), estudo, espiritualidade	Ofício, espiritualidade, esportes, educação e trabalho

Fonte: elaboração própria.

### Nove mulheres periféricas: panorama de uma minoria

As jovens entrevistadas apresentaram diferentes características do comportamento suicida/autolesão não suicida (Quadro 2), sendo reunidas enquanto um grupo feminino e periférico. Não há uma única minoria aqui representada, mas um grupo formado exclusivamente por mulheres nascidas e residentes em áreas periféricas de São Gonçalo. Por meio dessa exclusividade, reconheceu-se o caráter inclusivo da pesquisa, em que nove mulheres compuseram uma minoria marcada pela interseccionalidade ou, como definido por Akotirene ^36^, por um sistema interligado de opressões.

Baére & Zanello ^10^ debatem que os papéis de gênero numa sociedade patriarcal e violenta influenciam a saúde mental de mulheres, inclusive desqualificando o seu sofrimento emocional. No Brasil, o gênero feminino segue prevalente nos casos de tentativas de suicídio e autolesões não suicida, sendo o risco de suicídio mais elevado entre os 15 e 19 anos ^6^ ^,^ ^7^. Tais dados sinalizam a relevância dos aspectos socioculturais na compreensão da suicidalidade ^10^ e convergem à argumentação de Jaworski ^37^ de que, no gênero feminino, o foco investigativo deveria estar no início do espectro suicida e não em seu desfecho letal.

A descrição das jovens como periféricas e marginalizadas não se resume à renda ou classe social. Os territórios em que foram amostradas caracterizavam-se por contexto de intensa vulnerabilidade social; territórios marginalizados, periféricos e com alta criminalidade ^30^. Destaca-se que, apesar de possível convergência ou associação entre os conceitos marginalidade e criminalidade, apoia-se na diferenciação apresentada por Coelho ^38^ ao descrever o cenário de violência urbana no Rio de Janeiro, em que desemprego, subemprego ou pobreza seriam *proxys* de uma população marginalizada, elementos que, embora possam facilitar o surgimento da criminalidade, não a caracterizam per se. Ao mesmo tempo, as reincidentes operações policiais violentas que ocorrem em territórios periféricos ^32^ corroboram o processo da criminalização da marginalidade e da marginalização da criminalidade ^38^.

Desse modo, seguir residindo e se relacionando nesses territórios foi compreendido como uma permanência de imersão no cotidiano da violência e, logo, de vulnerabilidade no curso desenvolvimental ^4^ ^,^ ^19^. Assim, mesmo trabalhando e/ou escolarizadas, essas jovens estiveram à margem da sociedade, integrantes da desigualdade social, tendo o curso de suas vidas atravessado pela violência estrutural, emoldurando suas relações microssociais ^15^.

Foram analisadas três categorias: (1) trajetórias marginalizadas: visões sobre a violência autoinfligida; (2) palavras marginais: o quase dito sobre o comportamento autolesivo; e (3) à margem do suicídio: camadas de proteção e pontos de virada.

### Trajetórias marginalizadas: visões sobre a violência autoinfligida

Argumentos teóricos e clínicos apontam que a sobreposição de violências se torna um risco para a sua tipologia autoinfligida ^23^; decerto, isso não é especificidade das autolesões, mas uma condição frequente no estudo das violências ^28^. Nas interlocutoras, o relato de violência pode ser agrupado da seguinte forma: oito com violência familiar na infância e adolescência; quatro com violência na escola; duas com violência sexual; cinco com violência de parceiro íntimo.

Pela fala das jovens, as experiências pessoais marcantes da violência não justificaram a presença do comportamento suicida/autolesão não suicida em suas vidas. Isso surpreendeu inicialmente, uma vez que se reconhece as consequências das violências nas dimensões física, psíquica, desenvolvimental e relacional - marcadamente em crianças e adolescentes ^19^ ^,^ ^25^. A análise não descartou esse impacto, mas apontou para o não reconhecimento da violência em si. A explicação extrapolou a vivência pessoal dessas jovens, ampliando-se para percepções que expressaram os entendimentos sociais sobre tais fenômenos.

Dois núcleos explicativos permaneceram estáveis durante o processo analítico: “pecado” e “doença”. Ambos refletem o caráter desviante do suicídio na sociedade ocidental: deslizes individuais na/da trajetória humana ^12^ ^,^ ^39^. Houve gradações nessa categoria: “pecado” e “doença” mais facilmente se referem ao suicídio consumado. Sendo o desfecho mais trágico do *continuum* suicida, o suicídio foi apontado como “grande pecado” ou “intensa doença”; as autolesões não suicida como atos deliberados contra a própria integridade não representariam desvio tão grave como o suicídio.

“*A função do inimigo é matar, roubar, destruir. Então, ele quer que a pessoa se mate. Se a pessoa se matar, a pessoa não vai ter salvação!*” (Leci Brandão).

Houve um predomínio da perspectiva neopentecostal no sistema de fé e valores das interlocutoras. Quatro são neopentecostais praticantes, uma não praticante e uma “afastada”, todas com fortes marcas discursivas dessa doutrina. Mesmo nas duas jovens “sem religião definida”, notou-se influência do neopentecostalismo. Tal realidade corrobora os achados de Cunha ^40^, que aponta a capilarização desse segmento nas periferias do Rio de Janeiro, principalmente após os anos 1990, fortemente expresso na cultura, no cotidiano e na linguagem da juventude. Maia ^41^ também retrata esse contexto, descrevendo a visibilidade e o trânsito cotidiano dos jovens neopentecostais em São Gonçalo.

Desse modo, evidenciou-se como as crenças e as normas neopentecostais atravessaram as trajetórias dessas jovens, influenciando suas escolhas, moldando seus horizontes e explicando seu sofrimento e suas práticas autolesivas. Nessa cosmovisão, o mundo está em constante tensão entre o bem e o mal ^41^, sendo, segundo as entrevistadas, a ideação suicida uma influência maligna que as afasta dos caminhos e das promessas do Senhor.

“*Tem hora que somos falhos, somos pecadores. ‘Senhor, eu estou sem força!’. Então vem, vem uns pensamentos ruins na nossa mente. Nem toda hora a gente está ligado 100% em Deus!*” (Cora Coralina).

Pela visão do suicídio como pecado, o exercício da fé seria a principal intervenção a ser buscada diante do *continuum*, evitando que a ideação suicida chegue à forma de tentativa. A ideação ser vista como uma provação ou tentação que, ao ser superada por meio da fé, pode honrar a relação com o sagrado. Em contrapartida, um dos fracassos da espiritualidade ocorre diante do suicídio.

Uma estrutura religiosa que valoriza pregações simples, com exemplos simples para pessoas simples ^40^ teve grande aceitação nas periferias, facilitando a incorporação e a disseminação de seus princípios, como identificado neste *corpus*. Apoiado na teoria da prosperidade e do domínio ^40^ e na possibilidade de mudanças terrenas e não apenas no porvir ^41^, o neopentecostalismo se fortaleceu e assumiu uma dimensão considerável na compreensão de horizontes e trajetórias da juventude brasileira.

Admite-se, aqui, a pluralidade do segmento neopentecostal; nesta pesquisa, tais diferenças não são fundamentais, pois exprimem uma semelhante cosmovisão que auxilia a compreensão das micro e macrorrelações sociais nesse cronossistema ^15^, buscando sua incorporação como possível elemento nas redes de proteção. Acessar os espaços e as instituições da cultura neopentecostal, convocando-os como polos de cuidado e prevenção em saúde mental, gera inúmeros desafios e conflitos pelo contexto de violência e conservadorismo, mas deve ser planejado.

Foi consensual pelas jovens a explicação do suicídio como expressão de doença, entendimento fortemente amparado pela difusão do modelo biomédico. Tal paradigma justifica que em cerca de 80% a 90% dos suicídios consumados, há um transtorno mental associado, cifra replicada na literatura ^6^ ^,^ ^23^. Desse modo, evidenciou-se uma aproximação entre o senso comum e o conhecimento científico hegemônico.

“*É uma dor que você carrega, a pessoa acha que não, mas é um peso. Você dorme com isso, você acorda com isso; você não quer pensar, mas você acaba pensando. Isso te tortura de uma tal maneira. Eu costumo falar que eu não julgo quem quer tirar a vida*” (Elza Soares).

Tal horizonte não parece representar o esmaecimento dos tabus sobre o suicídio infantojuvenil ^41^, mas ser fruto de campanhas que abordam a prevenção do suicídio pela saúde. Uma campanha brasileira de prevenção ao suicídio foi citada por várias jovens, sugerindo sua incorporação no cotidiano, marcadamente pelas redes sociais. Essa incorporação, contudo, não necessariamente provoca mudanças individuais ou coletivas significativas para a prevenção, tendo uma presença por vezes indiferente ou tediosa, segundo elas.

O discurso das interlocutoras privilegiou, como defendido pela literatura biomédica, uma possível aproximação entre a depressão e o comportamento suicida ^1^ ^,^ ^2^ ^,^ ^23^, com exemplos de como o sofrimento psíquico pode facilitar a ocorrência dessa violência. Compreendeu-se que dois elementos facilitaram esse entendimento: a realização de psicoterapia por quatro das nove jovens; e narrativas cujo conteúdo, em certos momentos, sobrepuseram-se à descrição de intensa tristeza e desesperança. A investigação não era uma entrevista diagnóstica, mas o referencial teórico da suicidologia permitiu essas ponderações na análise.

Oito jovens sinalizaram sintomas compatíveis com transtornos mentais ao longo da vida; sendo que apenas quatro tiveram acesso à psicoterapia e nenhuma delas a tratamento psiquiátrico, havendo casos de uso irregular de psicotrópicos. Assim, a experiência pessoal de sofrimento emocional, ainda que não reduzida a um transtorno, pode se apresentar como um argumento para a correlação entre o comportamento suicida e o transtorno mental.

“*Uma situação mais de ‘cansei!’. Era uma sensação de: ‘Ah, não quero mais, não quero mais viver não. Cansei. Só dá problemas, só dá estresse, nada melhora, não vejo nada acontecendo, só dá tristeza’. Eu pensava que era mais fácil acabar*” (Clementina de Jesus).

É necessário demarcar o baixo suporte recebido por essas jovens diante de seu sofrimento psíquico. É possível que tenha sido reconhecido e não manejado, por um acesso dificultado às redes de atenção psicossocial, mas que, ainda mais grave, não tenha sido validado enquanto sofrimento. Alvarez et al. ^4^ apontam que apenas 1/3 da juventude negra norte-americana que morre por suicídio tem um transtorno mental diagnosticado. Baére & Zanello ^10^ e Jaworski ^37^ ilustram como a menor letalidade das tentativas de suicídio no gênero feminino pode ser compreendida socialmente como um menor sofrimento do que o encontrado no masculino. Navasconi ^11^, afastando-se criticamente da perspectiva biomédica, aponta que o racismo é fator de adoecimento e suicídio em jovens brasileiros. Nessa argumentação, apresentam-se autores diferentes, com perspectivas teóricas diversas que, para além de suas filiações, convergem sobre como o preconceito para com minorias deve ser considerado no manejo e na prevenção do comportamento suicida/autolesão não suicida.

### Palavras marginais: o quase dito sobre o comportamento autolesivo

Todas as entrevistas foram precedidas por uma breve explicação da investigação, identificando sua temática e motivação. Esse cuidado contribuiu na compreensão sobre a dificuldade observada para se falar na primeira pessoa e na utilização de palavras e termos diretamente relacionados ao comportamento suicida/autolesão não suicida.

Falar sobre terceiros, conhecidos ou não, foi constante, permitindo às jovens uma ampliação de argumentos, exemplos e explicações causais para as violências autoprovocadas. De igual modo, pareceu mais fácil se recorrer a metáforas ou ilustrações para narrar e exemplificar a própria experiência. Nesse ponto, justifica-se um salto compreensivo para além do expresso discursivamente, permitindo-se ouvir e interpretar o quase dito ou o silenciado ^33^.

“*Minha mãe me batia e eu queria sumir da vida dela. Eu não queria morrer, mas queria morrer pra ela, porque ela não ia mais me ver. Ela nem ia mais saber da minha existência. Eu queria sumir, quase morrer*” (Clara Nunes).

“*Sei lá, acho que vontade de se matar nunca tive. Agora de sumir assim, se esconder, entrar em um buraco e ficar ali escondida, sem ninguém me ver. Sumir e, se tivesse uma solução, eu voltava*” (Conceição Evaristo).

“*Eu me via como se eu tivesse deitada numa maca no hospital. Eu deitada lá, via o médico, mas eu não via ninguém da minha família. Eu ficava desacreditada. ‘Gente, será que é isso mesmo? Será que ninguém? Não é possível!’ Aí via que ninguém aparecia. Ficava um pouco nervosa, estressada, pensativa*” (Elza Soares).

Mais do que a identificação e até mesmo a compreensão do quase dito pelas jovens, abarcar a marginalidade dessas palavras incorporando-as à semântica do cuidado é uma das possibilidades desta pesquisa. Ponderou-se que a cristalização do tabu sobre o suicídio infantojuvenil ^42^ pode ter sido um motivo para evitar o assunto. Tabu que interdita a temática, silenciando e constrangendo o discurso. Porém, o percurso pessoal narrado em paralelo à notoriedade contemporânea dada ao tema sugere que outros elementos possam esclarecer as palavras marginais.

Amparando-se por estudos que apontam para a discriminação contra minorias como fator de risco para seu comportamento suicida/autolesão não suicida ^19^ ^,^ ^20^, considera-se que a não validação social do sofrimento psíquico ao longo da vida dessas jovens seria um sensível e pertinente entendimento para essa apreensão empírica. Uma não validação ou ainda uma não adequação de um mal-estar, que só é reconhecido, acolhido e manejado se nas cores e formas hegemônicas, como discute Fanon ^21^.

Ao apontar um maior número de tentativas de suicídio do que de relatos de ideação suicida em jovens negros, Benton ^5^ corrobora que o sofrimento dessa minoria só é validado diante de ações mais drásticas. Como se a grupos minoritários o sofrimento psíquico fosse naturalizado e interditado, não ouvido. Ao mesmo tempo, Jaworski ^37^ salienta que o menor número de suicídios globalmente em mulheres não deve mascarar a presença de ideação suicida nesse gênero, alertando para outros formatos de discriminação.

Sheftall et al. ^9^ defendem que as violências estruturais e relacionais precisam orientar as investigações, abordagens e ações de prevenção da suicidalidade em juventudes minoritárias. No Brasil, Navasconi ^11^ denuncia como a literatura científica e as ações em saúde ignoram a interseccionalidade do comportamento suicida com marcadores de raça, classe e gênero.

Sendo as diferentes discriminações e violências uma realidade presente nas relações sociais de grupos periféricos, principalmente os femininos, é essencial que a escuta sobre a suicidalidade considere as adversidades ambientais e as experiências adversas na infância ^19^; uma escuta sensível e um olhar pelas lentes da justiça, da equidade, da diversidade e da inclusão ^20^ ^,^ ^36^. As violências reiteradas e naturalizadas para com esses grupos se comportam como microagressões cotidianas, impactando sua saúde mental principalmente quando silenciadas ou ignoradas ^27^.

Nesse horizonte, o discurso mais distante das jovens entrevistadas sobre comportamento suicida/autolesão não suicida não pareceu expressar somente um desconforto diante de uma entrevista. As palavras quase não ditas revelam o apagamento de um sofrimento. Ou, ainda, delatar perdas de oportunidades de acolhimento diante de diferentes violências (Quadro 2) nos sistemas relacionais ao longo das trajetórias de vida.

### À margem do suicídio: camadas de proteção e pontos de virada

Essa categoria permitiu o reconhecimento de elementos do cotidiano das jovens (funções sociais e/ou intervenções) identificados como camada de proteção para o suicídio (Quadro 2). Tais elementos foram agrupados em oito categorias de primeira ordem e interpretadas e reclassificadas em três de segunda ordem ^33^. As três categorias - espiritualidade, ofício e maternidade - foram percebidos como os pontos de virada mais relevantes vivenciados pelas jovens. Tal síntese facilitou um olhar sobre os contextos que permitiram uma trajetória à margem do suicídio, apesar do comportamento suicida/autolesão não suicida ao longo de suas vidas.

Destacar os pontos de virada foi também um recurso metodológico utilizado por Werner & Smith ^43^ numa coorte de crianças havaianas (Estados Unidos), iniciada na década de 1950. As autoras buscaram compreender, na fase adulta, quais elementos cotidianos foram os protetores longitudinalmente. Educação Superior; ofícios aprendidos no serviço militar; casamento estável; participação numa comunidade de fé; recuperação de um evento ameaçador de vida; e psicoterapia foram os pontos reconhecidos na coorte havaiana. Alguns são semelhantes aos encontrados nesta pesquisa.

O debate prévio sobre a influência da dimensão espiritual na vida das jovens justifica a importância da categoria “espiritualidade” como um elemento de proteção e resiliência. E, mais do que uma ponderação ontológica, sua relevância está em sua concretude ordinária; a espiritualidade promove a organização e a inclusão em uma rede de apoio no cotidiano real. Grupos orgânicos que se encontram, validando os valores e o esforço por uma vida melhor ^40^. A escolha individual por uma inserção religiosa na adolescência demonstrou maior apelo como apoio social do que quando representado pela continuidade da herança religiosa parental ^41^.

Ofício, nesta pesquisa, ultrapassa o trabalho. Por capacidade laboral ser um parâmetro de avaliação do transtorno mental e da suicidalidade, enquanto desvios da ordem social ^39^, evitou-se essa dimensão mais fortemente provocada pelo termo “trabalho”. A categoria ofício agregou outras intervenções e camadas de proteção (Quadro 2): artes (curso para ensinar dança), prazer em trançar (curso para o ofício de trancista), escolaridade e prática laboral em si (inserção na teia social). As jovens legitimam que a capacidade - obtida ou desejada - de se sustentarem confere-lhes segurança e dignidade. Como mulheres periféricas, a busca pela independência financeira é um elemento evidente de proteção - diante de violências e do sofrimento emocional, principalmente para as que têm filhos.

A escolarização, um elemento formador dessa categoria, foi ponto criticado, mesmo que apenas duas jovens não tenham concluído o Ensino Fundamental. A escola não se mostrou como um espaço de formação adequada para seu cotidiano e não conseguiu garantir acolhimento, estabelecendo-se como um ambiente violento. Diante do comportamento suicida/autolesão não suicida, predominaram relatos que sinalizam uma incapacidade de diálogo e manejo. Nesse horizonte, Sheftall et al. ^9^ debatem que jovens negros tendem a se sentir menos seguros em relação à saúde mental na escola, não buscando auxílio como seus colegas. A presença de dispositivos escolares de ajuda não atinge a todos, demandando estratégias mais sensíveis à subjetividade de grupos minoritários. Os autores ^9^ apostam que o treinamento de lideranças de associações comunitárias e organizações religiosas na abordagem da violência, no manejo de conflitos interpessoais e no reconhecimento do sofrimento emocional poderia diversificar, de forma contextualizada e decolonial, as estratégias de prevenção e abordagem do comportamento suicida/autolesão não suicida, dando voz e protagonismo às identidades historicamente silenciadas e desautorizadas, como argumenta Ribeiro ^44^.

A maternidade foi um ponto cotidiano importante: das nove jovens, seis eram mães, estando uma em sua terceira gestação; das três jovens sem filhos, uma evidenciou seu desejo pela maternidade. Num primeiro olhar, a presença da maternidade poderia expressar a perpetuação do papel feminino na procriação, desejada ou não, ou ainda ser uma escolha para essas jovens, mas essa não foi a realidade encontrada. Para algumas interlocutoras, a gestação atravessou negativamente o curso da vida, justificando a evasão escolar, ampliando as violências e agravando o sofrimento psíquico.

Diante da provocação de como a maternidade influenciou o curso do comportamento suicida/autolesão não suicida, todas concordaram que a gestação e o nascimento dos filhos foram elementos de proteção, mesmo àquelas que referiram sofrimento e violência. Segundo as jovens, a maternidade foi principalmente uma possibilidade de se recontar a própria história, repensar valores e atitudes: uma experiência de mudança. Questionou-se a tonalidade romântica nessa perspectiva, evidenciando-se que, ao se tornarem mães, os filhos se tornaram sua maior preocupação. Os filhos, especificamente as filhas, precisam ser protegidas das violências experimentadas por elas. Um grande empenho é necessário, levando a um planejamento de vida que as afastou da suicidalidade.

“*Eu tenho uma filha. Eu preciso mostrar pra ela que o caminho não é esse. Eu não preciso levar a minha vida para o ralo, porque se não a minha filha também vai levar a vida dela pro ralo. Imagina isso acontecer?*” (Leci Brandão).

Não se trata de uma defesa de que a maternidade seja universalmente protetora, até pela possibilidade de agravamento de violências e transtornos mentais ^23^. Mas é importante reconhecer que a maternidade pode permitir reorganizações nas relações sociais e proporcionar esperança; um horizonte esperançoso é um ponto de proteção ^8^. Ao mesmo tempo, devem ser demarcadas as preocupações transgeracionais com a violência de gênero ^10^, com um maior temor com o futuro das filhas do que dos filhos.

“*Essa pessoa aqui é guerreira, luta pelas filhas, mas a vida diária é tristeza, virou uma tristeza, coisa que eu não consigo nem falar. Eu fico olhando assim no espelho e fico assim falando: ‘Por que eu estou chorando? Por que eu estou triste? Tenho minhas filhas, tenho minha casa!’*” (Elza Soares).

Salienta-se que os pontos de virada podem ser concebidos não apenas como fatores ou camadas de proteção, mas como uma associação entre esses elementos de resiliência ^43^, importantes por sua especificidade e historicidade, conforme o modelo bioecológico ^15^. Defende-se que a compreensão sobre a suicidalidade e o planejamento de intervenções psicossociais considerem as trajetórias de vida, a comunidade e os elementos significativos do cotidiano dos sujeitos e seus pares ^11^. A aproximação crítica com o espaço comunitário não é aqui colocada como inovação, considerando sua nuclear defesa pela Saúde Pública. Reconhece-se, contudo, a insuficiência dessa prática pela suicidologia, principalmente diante de minorias, cujas existências marginalizadas tendem a ser menos contempladas e compreendidas pelos campos de conhecimento e por políticas públicas, como provocado por Lima & Navasconi ^13^ e Ribeiro ^44^.

## Considerações finais

Neste artigo, pensou-se sobre a vida por meio de experiências de suicidalidade, relatadas por jovens periféricas. Vida e morte num diálogo entremeado por sofrimentos e violências que marcam a subjetividade e o horizonte de uma juventude à margem da sociedade. Acionar esse sofrimento a partir de vivências fronteiriças com a morte (ideação e tentativa de suicídio) e autolesões não suicida permite um olhar dinâmico para o risco e, mais importante, para a proteção desses grupos minoritários, cujo sofrimento e cujas linguagens tendem a ser menos validados, recebendo menor suporte e intervenções psicossociais. Esse olhar também denuncia o impacto da violência de gênero no horizonte relacional das mulheres.

Explorou-se a realidade de mulheres periféricas, assumindo-as enquanto grupo minoritário para refletir sobre comportamento suicida/autolesão não suicida; o debate, entretanto, ampliou-se para além do gênero e da classe social. Há riscos numa argumentação que sobrepõe diferentes minorias e isso é uma limitação desta pesquisa, que pode, porém, potencializar a discussão contra-hegemônica e interseccional sobre a suicidalidade na juventude do sul global. Investigações que aprofundem a relação do comportamento suicida com a violência urbana e a juventude masculina, ou ainda sobre as nuances das minorias étnico-sexuais, podem dialogar complementarmente com as reflexões aqui apresentadas. Outra potencial limitação foi que a informação sobre comportamento suicida/autolesão não suicida nas primeiras ondas da coorte foi obtida com responsáveis e professores, embora esses sejam importantes informantes da pesquisa em saúde mental infantil.

Diversificar cenários clínico e epidemiológico deve estar na pauta das novas pesquisas em suicidologia, numa crítica urgente para o reconhecimento de que a universalidade do suicídio não é composta apenas por grupos majoritários e hegemônicos. O humano e o universal são recheados de minorias, em suas especificidades e complexidades; reconhecer isso pode tornar a meta da redução da morbimortalidade do comportamento suicida na juventude mais factível. Estreitar olhares, aprofundando-os sobre grupos violentados, não apenas qualifica o cuidado e a prevenção do comportamento suicida nessa população, mas amplia o entendimento desse fenômeno. Para isso, entretanto, é preciso reconhecer a humanidade, os direitos e a saúde mental de grupos historicamente vulneráveis e discriminados.

Ao mesmo tempo, pensar sobre a resistência da vida e desses corpos femininos é reconhecer que há estratégias, para além das biomédicas, de proteção. As camadas de proteção e os pontos de virada se estabelecem no cotidiano ordinário da juventude, onde e com quem transitam, se relacionam, exercem suas crenças e vivem seus desejos e labor. Transformar esses contextos em “fatores de proteção” pode significar uma intervenção através das lentes de equidade, interseccionalidade e inclusão, extrapolando as estratégias individuais às estruturais. Assim, a prevenção da violência autoinfligida precisa validar, numa prática ética e decolonial, as subjetividades, os saberes e os sofrimentos apagados e negligenciados.
